# Multi-Filter Decoding in WiFi Backscatter Communication

**DOI:** 10.3390/s21041481

**Published:** 2021-02-20

**Authors:** Richard Boateng Nti, Ji-Hoon Yun

**Affiliations:** Department of Electrical and Information Engineering, Seoul National University of Science and Technology, Seoul 01811, Korea; richardnti@seoultech.ac.kr

**Keywords:** WiFi backscatter communication, multi-filter design, frequency-shift, IoT

## Abstract

WiFi backscatter communication has emerged as a promising enabler of ultralow-power connectivity for Internet of things, wireless sensor network and smart energy. In this paper, we propose a multi-filter design for effective decoding of WiFi backscattered signals. Backscattered signals are relatively weak compared to carrier WiFi signals and therefore require algorithms that filter out original WiFi signals without affecting the backscattered signals. Two multi-filter designs for WiFi backscatter decoding are presented: the summation and delimiter approaches. Both implementations employ the use of additional filters with different window sizes to efficiently cut off undesired noise/interference, thus enhancing frame detection and decoding performance, and can be coupled with a wide range of decoding algorithms. The designs are particularly productive in the frequency-shift WiFi backscatter communication. We demonstrate via prototyping and testbed experiments that the proposed design enhances the performance of various decoding algorithms in real environments.

## 1. Introduction

From unmanned aerial vehicles (UAVs) to smart homes, smart factories, smartphones and many more, Internet of things (IoT) brings together data, processes, people and things all networked to produce an immense infrastructure of information system. Technological advances in IoT open a wide range of applications that call for hundreds of billions of sensors communicating. One critical challenge towards the vision of IoT is to build devices that can be easily deployed and run autonomously for a lengthy duration [1,2]. Backscatter communication offers a promising remedy as to a core technology for IoT applications, harvesting wireless power from and transmitting data on received radio signals rather than generating its own.

Backscatter communication is simply reflecting and modulating an incident radio frequency (RF) signal. A backscatter tag switches between reflection and absorption states by impedance tuning. Over the past years, two types of backscatter communication exist: monostatic backscatter and bistatic backscatter. Typical applications of monostatic backscatter communication are point-to-point communication in the RFID tag where the transmitter and receiver are the reader communicating with a tag. Only two devices are involved in conventional RFID leading to a limitation in distance (range), data rate, modulation scheme, point-to-point networking and a dedicated energy source. Ambient backscatter [3,4], a bistatic backscatter communication with a separate transmitter, receiver and a tag, is promising due to the use of ambient RF signals [5], for example, FM radio [6], TV signals and WiFi signals [7,8], toward communication channels rather than a dedicated reader or infrastructure.

IEEE 802.11 popularly known as WiFi is more or less built into almost all modern-day electronic gadgets from PCs, laptops, smartphones to gaming devices and even running shoes. Abundance creates a solid platform for WiFi backscatter communication. WiFi backscatter is a type of ambient backscatter that utilizes only WiFi signals as its carrier signal. This plausible concept leverages the possibility to reuse existing WiFi infrastructure to provide connectivity relying on ambient WiFi as its excitation signal. As a result of its attractive features, WiFi backscatter has seen developments and prototyping lately from the industry and academia [9,10,11,12,13,14].

A critical observation on the trend of WiFi backscatter reveals a paramount issue which is the severe fluctuation of WiFi signals due to the nature of orthogonal frequency-division multiplexing (OFDM). Such fluctuation remains not only in backscattered signals but equally in carrier WiFi signals known as self-interference, making decoding a challenging task. Since WiFi signals have a much shorter symbol duration (e.g., 4 s) than backscatter signals, the fluctuation can be smoothed out to some degree by a low-pass filter. Therefore, low-pass filtering of received signals is essential prior to decoding in WiFi backscatter communication. However, designing a filter dedicated to WiFi backscatter has been unexplored in the literature.

Frequency-shifted backscatter (FS-backscatter) [15] can also be considered to avoid the self-interference associated with WiFi backscatter. The central idea of FS-backscatter is to shift the WiFi signal prior to reflection to a non-overlapping adjacent frequency channel. However, extraction of data from raw signals from frequency-shift is relatively challenging due to its low signal-to-noise ratio (SNR); thus, the signal amplitude is close to the noise floor. Therefore a filter, required to separate signals from noise, is still essential to decoding the received signal.

In this paper, we proposed a multi-filter design for decoding in WiFi backscatter communication with frequency shift, which can be combined with a wide range of existing decoding algorithms for performance enhancement. We propose two multi-filter schemes—summation and delimiter—and a hybrid of the two with engaging the use of more filters. The placement of filters and evaluation of signal processing account for the difference in the design methodology. The utilization of multiple filters in our proposal allows us to repeatedly remove unwanted signals with different filter parameters and efficiently identify packets. As a result, it becomes easy for the determination of a threshold relevant for decoding. The proposed schemes accommodate for changes in amplitude fluctuation as well as the needless effort to preview the raw signals severally before setting the threshold.

We demonstrate via our prototype implementation and testbed experiments in office and hallway scenarios that the proposed designs outperform the conventional single-filter decoding in various conditions. The margin of BER decrease reveals a huge performance difference between the single filter design to the tune of 34% and 44% enhancement for our multi-filter summation and delimiter schemes, respectively, under a good channel condition. On the other hand, i.e., in worse channel conditions, the maximum BER improvement shows approximately 98.5% and 97% for summation and delimiter multi-filter designs, respectively, attained over the single filter design.

In summary, the contributions of this work are as follows:

- Experimental observations that reveal the high sensitivity of signal shape to the configuration of a preprocessing filter.
- Design of multi-filter schemes for effective noise isolation for reliable decoding in WiFi backscatter communication with frequency shift implementation.
- Prototyping and a testbed experiment performed to verify the validity of the proposed multi-filter designs in real-world environments.

The remainder of this paper is sectioned as follows: Section 2 talks about the related works while Section 3 looks at the system model of the building components. Section 4 throws light on experimental observation leading to the motivation of this work and Section 5 describes the proposed dual-filter design. Section 6 examines the performance evaluation of results while the concluding remarks are given in Section 7.

## 2. Related Work

The research community has published a rich body of papers on backscatter communication [4,5,15,16,17,18,19,20,21,22,23,24,25]. Signal detection in ambient backscatter communication can be problematic. Ref. [4] developed a theoretical model for the tag with a differential encoder to eliminate channel estimation. The authors in addition derived the closed-form threshold for optimal detection and minimum-BER detector. The authors in [5] proposed WiFi backscatter with the intention to prove the reuse of existing WiFi infrastructure to provide internet connectivity. The paper aimed to enable RF-powered devices to communicate with off-the-shelf WiFi devices. First, the paper considered uplink communication and designed a system that permits WiFi backscatter tags to convey information to WiFi devices. To achieve this, the system modulated the WiFi channel information with the inclusion of received signal strength indicator (RSSI) and channel state information (CSI). On the other hand, the authors sought to overcome the downlink communication challenge of a reader only enabled to transmit WiFi packets. A circuit for the backscatter tag was designed to detect RF energy to decode information.

BackFi [17] proposed a system of WiFi backscatter targeting long-range communication and high throughput connecting WiFi access point (AP) and IoT sensors. They developed an IoT sensor for backscattering WiFi signals and a radio circuit as well as an algorithm for the WiFi AP which doubles as a reader and a standard WiFi transmitter. Consequently, BackFi improved backscatter decoder by using a wideband signal instead of toning an excitation signal. Ref. [14] proposed multiscatter, a backscatter protocol for personal IoT sensors implying multiple excitation signals like WiFi, ZigBee and Bluetooth can work concurrently while maintaining ongoing data communication for legacy wireless technologies. In addition to conveying tag information on multiple signals, an overlay modulation, i.e., a new modulation scheme, was employed and yet ultra-low power consumption was achieved. Implementation was carried with a field programmable gate array (FPGA) and commodity radios for ZigBee, Bluetooth and WiFi, respectively.

The novel approach of FS-backscatter is from the article [15]. The basic idea was to separate the spectrum of the backscattered signal from the spectrum of the carrier signals by shifting to a cleaner adjacent channel for efficient decoding. Due to less interference experience in the clean channel using FS-backscatter, communication with commercial WiFi and backscatter tag could reach a longer communication range. To improve robustness, the authors leverage the plethora of radios available on smart devices to construct multi-carrier scenarios. An extension of the initial work on FS-backscatter published by [15] deploys an implementation making use of full commodity WiFi infrastructure by HitchHike [18] and FreeRider [19]. The FS-backscatter tags were designed to reflect entirely on 802.11b packets from off-the-shelf WiFi transmitter and a commodity 802.11b receiver can decode backscatter signal as standard WiFi packet. The basis of the article was codeword translation providing backscatter tag the ability to alter an original 802.11b codeword to another valid 802.11b codeword. A straightforward XOR operation supported in the design of the efficient decoder allowing piggybacking of backscattered data on transmission. Another decisive contribution of HitchHike was the cancellation of one sideband created during FS-backscatter.

The authors in [21] design a multi-antenna backscatter transmitter namely MO, and a low-power coding mechanism known as code, to boost the communication performance in terms of transmission range and data rates. By the application of multiple antennas, the authors eliminated interference from ambient RF signals such as TV signals, hence increasing the bit-rate among the transmitters in backscatter communication. Recently, MOXcatter [10] explores features in spatial multiplexing; thus, Wi-Fi backscatter operates with spatial streams with commodity radios without affecting productive data communication. MOXcatter allows multiple spatial stream packets to be embedded and can be decoded on COTS WiFi devices. A pattern-based decoding algorithm was proposed by [22] that identifies unique patterns of signal samples that result from the smoothing of WiFi signals to filter noise due to fluctuations. The new decoding algorithm operated differently in classifying the amplitude levels of the received signals, slope patterns were utilized consequently making the algorithm more robust against the configuration of the threshold.

## 3. System Model

**Figure 1:**
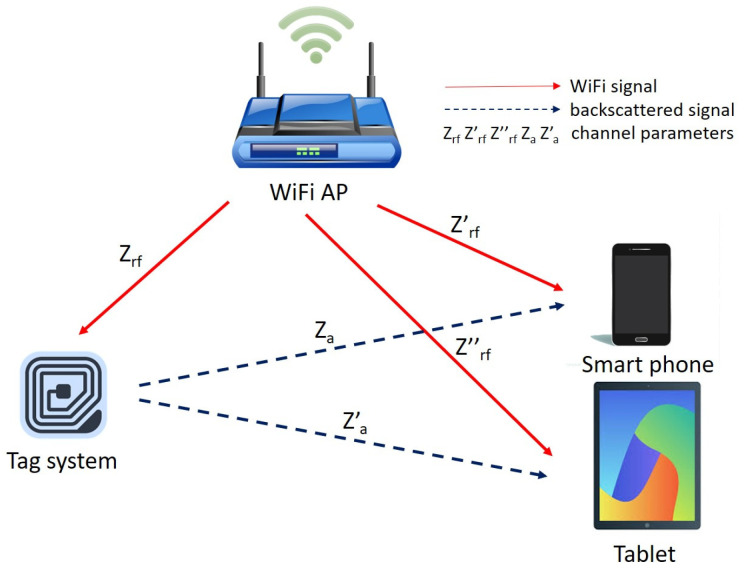
System model.

The communication system model presented for this paper is illustrated in Figure 1. Bistatic backscatter communication is modeled as consisting of a separate transmitter, receiver and a tag system. The transmitter in this case is a WiFi AP equipped to work with the 2.4 GHz frequency band. The receiver on the other hand can be any computing device, such as a laptop, smartphone, tablet, etc., with WiFi capabilities. It is imperative to stress the importance of WiFi because the system operates by piggybacking information on an existing WiFi signal, as such extraction of data for digital processing requires more-or-less basic protocols like channel categorization. The equation for the principle follows below with s(t) as the WiFi carrier signal and b(t) as the binary information bit conveyed, either zero or one and y(t) represents received signals:

(1) $$y\left(t\right)=Z_{rf}^{\prime }s\left(t\right)+r\left(b\left(t\right)\right)Z_{rf}Z_{tag}Z_{a}s\left(t\right)+\sigma$$

where $Z_{rf}^{\prime }$ and $Z_{rf}$ are the channel gains from the carrier source to the receiver directly and the tag, respectively; $Z_{a}$ is the channel gain from the tag to the receiver (reflection path); $Z_{tag}$ is the tag’s reflection gain and σ is the noise floor. In the equation, r(·) represents the switching state of the tag which depends on what information bit the tag transmits. We assume that the tag reflects signal (r=1) for data bit one, and this results in an increased (high) amplitude level of the backscattered signal while it absorbs the signal (r=0) for data bit zero, and the backscattered signal has no increased (low) amplitude level. Without frequency shift, a threshold to classify the information bit between zero and one should be configured between $Z_{rf}^{\prime }s\left(t\right)+\sigma$ and $Z_{rf}^{\prime }s\left(t\right)+Z_{rf}Z_{tag}Z_{a}s\left(t\right)+\sigma$. With frequency shift, it should be configured between σ and $Z_{rf}Z_{tag}Z_{a}s\left(t\right)+\sigma$.

**Figure 2:**
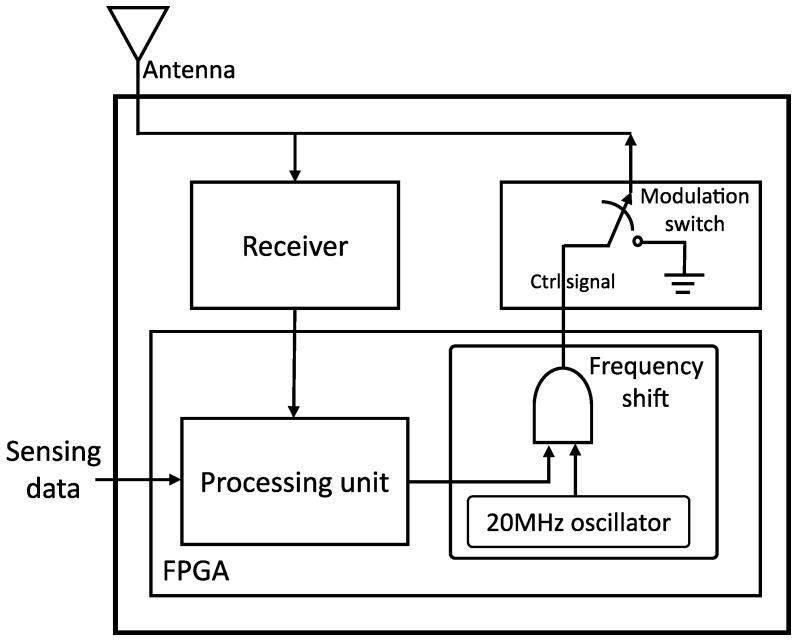
Tag system with frequency-shift.

The tag system in the model dictates the actual operation of the backscatter communication, thus reflection, modulation and frequency shifting. Notice the inclusion/addition of an oscillator shown in Figure 2 to shift backscattered signals to 20 MHz adjacent channels, i.e., 20 MHz behind the center frequency and 20 MHz beyond the center. This system essentially consists of a controlling element, i.e., FPGA fed with sensed data to generate control signal connected to the tag switch. The FM0 [3] coding technique, which is also called biphase space encoding, was used for generating alternating zeros and one’s bit information. The coded-bit rate of FM0 is a double of the information bit rate because a transition may happen in the middle of a bit. For the logic value of one, the bit is maintained from the center while logic zero experiences a transition in the center of the bit. It is known to minimize transmission power and reduce noise.

## 4. Observation on Preprocessing of the Received Signal

In this section, we take a look at two major points that serve to initiate the essence of this paper. We first observe from the experiment the impact of threshold to eliminate unwanted signal fluctuations and finally the effect of moving average filter in regards to the length.

### 4.1. Threshold Defined Segregation

The application of a filter in digital signal processing targets both the separation of combined signals and signal restorations distorted in some way. In this instance, the filter is employed as a low-pass filter implemented with moving average. However, after the separation, certain unwanted signal fluctuations or interference signals are propagated through to the output. The ability to remove such fluctuation or interference significantly affects the frame identification leading to the deletion of such signals. A threshold elevated above those signals will do the work but determining the accurate value is difficult especially in frequency-shift where the actual signals are close to the noise floor.

**Figure 3:**
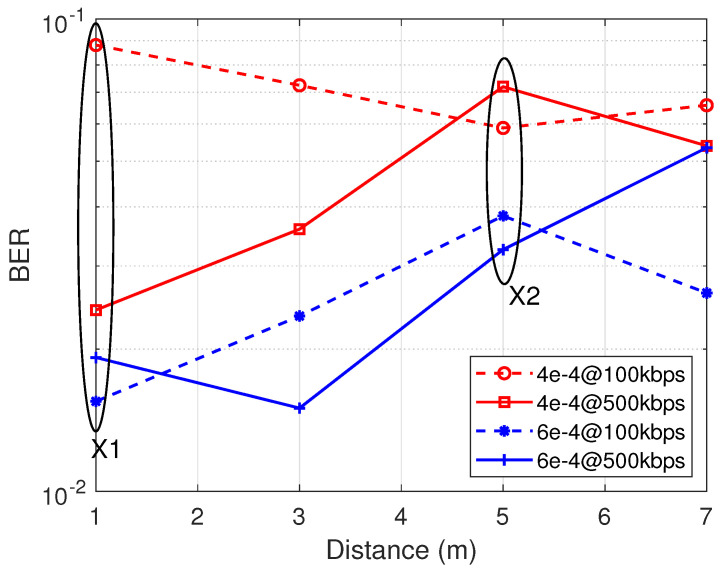
Fixed threshold instability with minimal difference.

Traditionally, a trial-and-error approach is adopted to find the appropriate threshold value. The above-mentioned appropriate threshold value, even though it comes with many challenges, works when the suitable value is determined. However, determining the correct threshold can be time-consuming and unstable when that fixed threshold moves through a fluctuating signal. From experimental observation, it can be seen that, a very small change in threshold causes the performance of the system to behave differently. Figure 3 illustrates a graph with two fixed threshold values: 4 $\times \;10^{-4}$ and 6 $\times \;10^{-4}$. At point X1 with a tag-to-receiver distance of 1 m, a significant gap is seen between 4 $\times \;10^{-4}$ for 100 kbps and 4 $\times \;10^{-4}$ for 500 kbps which contradicts the normal trend of higher data rate equivalent to higher BER. Thus, BER of 500 kbps is far better than BER of 100 kbps, veering from standard knowledge. On the other hand, the values for 100 kbps and 500 kbps at threshold 6 $\times \;10^{-4}$ regarding X1 provide a valid evaluation. Moving forward to the point labeled X2 in Figure 3, the direct opposite witnessed at point X1 is depicted in that that threshold at 6 $\times \;10^{-4}$ deviates from the norm while the threshold at 4 $\times \;10^{-4}$ follows an expected trend. The plot is a clear indication of inconsistency with fixed threshold. In conclusion, it can be said that a marginal value change in the threshold could cause the performance in error detection to deviate. This calls for a better way to evaluate threshold determination to minimize the margin of error as much as possible.

### 4.2. Filter Window Length

The moving average filter has widely been used because of its simplicity, efficiency in noise reduction and a good step response in the time domain [26,27,28]. By means of averaging a defined number of points, the moving average is implemented by convolution from a given input signal to a resulting output signal. The equations below explain the design parameters:

(2) $$\begin{array}{c} y\left[k\right]=\frac{1}{N}\sum_{i=0}^{N-1}x\left[k-i\right], \end{array}$$

where y[k] is the current output, x[k] is the current input, x[k−1] is the previous input and *N* is the length of the average or number of points.

**Figure 4:**
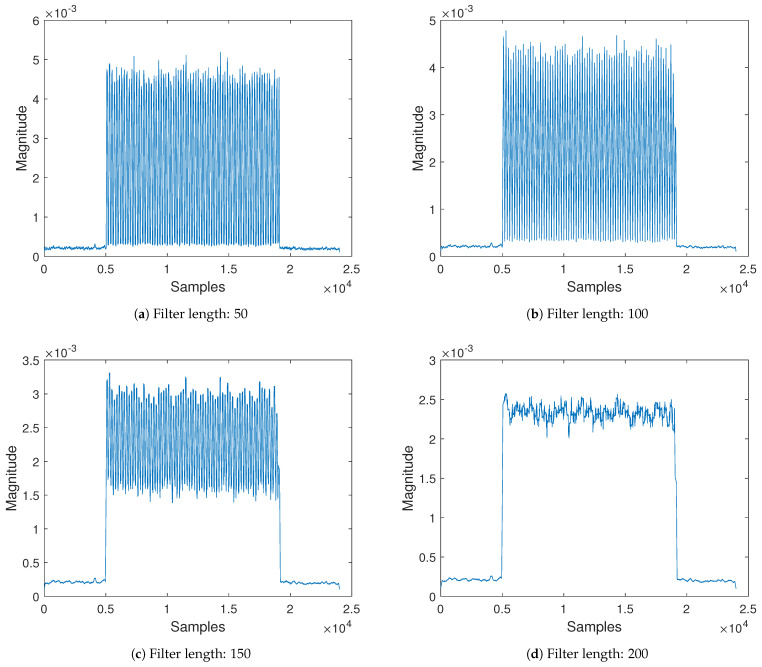
Moving average filter response with increased filter length.

The key parameter of interest to us in the equation is the value of *N*. Figure 4 gives an illustration and effect of different filter lengths. As *N* increases, there is more smoothing action, i.e., noise reduction evident from the amplitude. However, the sharpness of the edge becomes more blunt. It is worthy to note that both features of smoothing and sharp edge have technical benefits that can combine to achieve enhanced performance. It is shown in the figure that *N* affects how these features appear in the filtered signal. In summary, we seek to explore the merits of each side to improve the BER of frequency-shift backscatter decoding by exploiting multiple filters with different *N* values. It is also noted that *N* also affects the level of an appropriate threshold for bit decoding. This is because as shown in Figure 4 different *N* values result in various amplitudes of the filtered signal. A critical observation can be witnessed when the multiplying factor of the window length is five causing over-smoothing.

To address the above observations, the following questions are primarily considered:

1. How many filters are needed?
2. What length of filter *N* is optimal?
3. How is the threshold determined for efficient operation?

## 5. Multi-Filter Decoding

**Figure 5:**
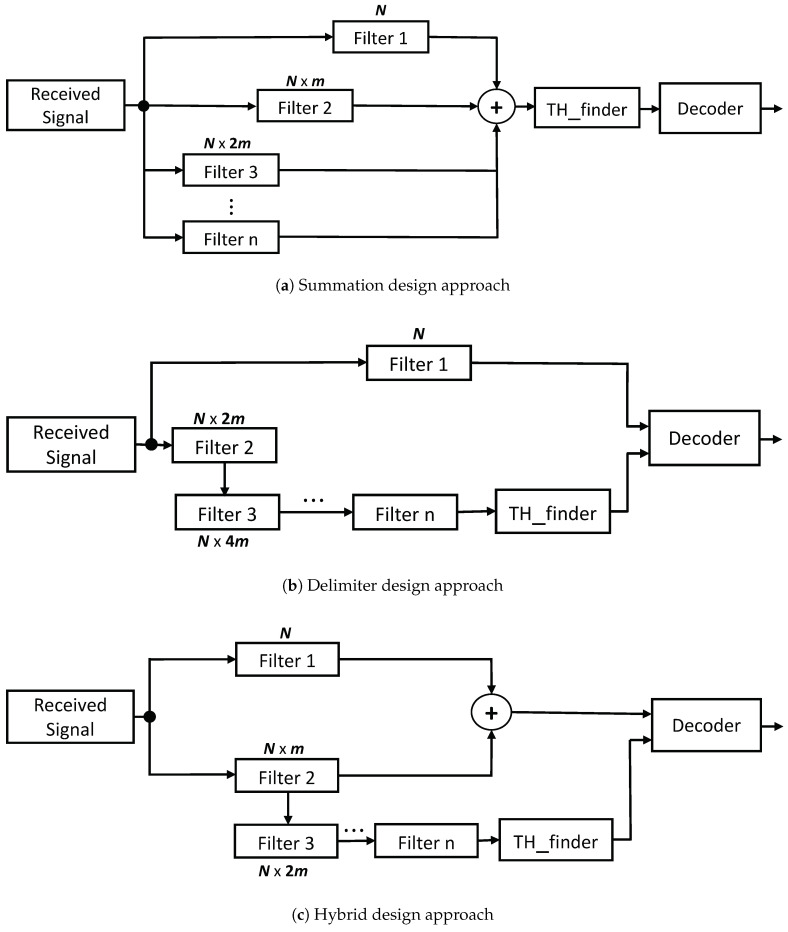
Proposed architecture of the multi-filter design.

In this section, we propose multi-filter designs as low-pass filters for efficient fluctuation/interference suppression before decoding. Convolution operation easily computes moving average filtering [26,28]. As such, for noise reduction and smoothing in addition to its ease of applicability and efficacy, a moving average filter was adopted. Furthermore, a moving average filter is well suited for time domain signal processing [26,27]. The observation from Section 4 necessitated the exploration of utilizing multiple filters for adequate noise separation and retaining a good step response. In regard to this, we propose two multi-filter designs with different architectures and a hybrid of two to effectively and efficiently capture the signal of interest. Figure 5 shows the designs of our proposed multi-filter approaches.

### 5.1. Summation Approach

**Figure 6:**
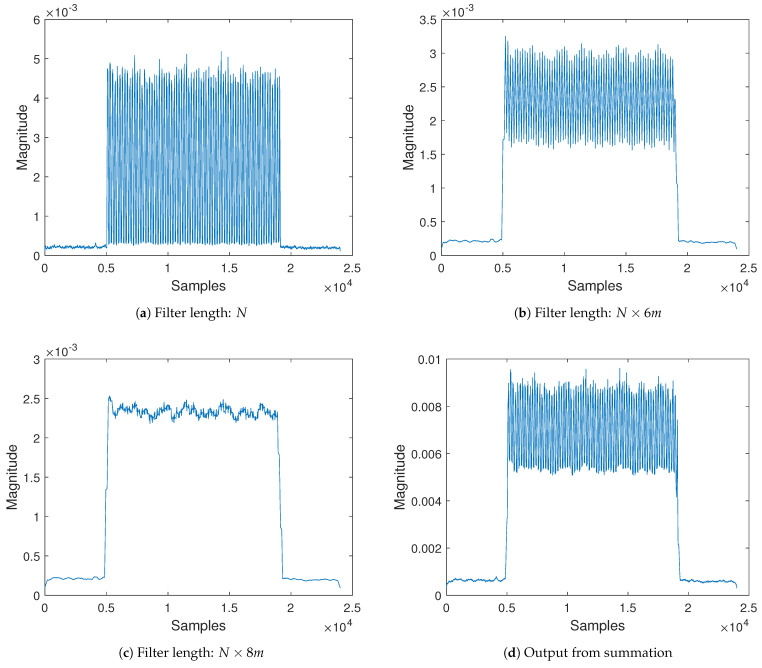
Filtered signal design of the summation scheme.

The summation approach depicted in Figure 5a is straightforward and simple yet efficient. The idea is to combine multiple filtered signals from the same source but with different filter parameters. The filter with length, *N*, i.e., Filter 1, produces an output coded with a significantly good amount of information although separation from the noise floor is difficult. The introduction of multiple filters with the length of N×m, where *m* is an integer greater than one, results in loss of coded information due to smoothing but with relatively elevated signal from the noise floor. A pivotal examination from Section 4 revealed a feature that when the multiplication constant of the filter is a multiple of four, over-smoothing happens. The over-smoothing effect produces a far less amount of information, however it causes step up distance above the ground floor indispensable for decoding. Therefore, the summation approach selects at least one filter with a constant multiplier of four and other filters with much better signal quality. Adding the three outputs from the filters reduces the quality of coded information but keeps it distinctively separable from the noise floor. Therefore, threshold can easily manage the removal of the unwanted noise floor signal. Illustration of the above-explained design can be seen in Figure 6. Whereas Figure 6a has a filter length of 50, Figure 6b,c produce a filter length of 300 and 400, respectively. The output from the three filters results in Figure 6d equipped with a good amount of information and clearly off the noise level. The output after the threshold finder is directly fed into the decoder for post-processing.

### 5.2. Delimiter Approach

The structural design of the delimiter approach shown in Figure 5b, unlike the summation multi-filter design, consists of cascaded filters with N×4m, N×8m filter length. Whereas in the summation approach the goal was to elevate the signals for separation, the delimiter approach seeks to maintain the amplitude of the original signal from the filter with length *N*, but use the other filters to uniquely identify the frames. The margins or edges of the filter with length N×4m act as boundaries that cut off or segregate the noise from the desired signal of filter 1. Bear in mind only filter lengths with a multiple of 4 are required in the delimiter approach; the reason being that signal degradation caused by over-smoothing has no ill-effect on the design. The concept is viable because when the filter length is increased reasonably, though the data become blunt (signal quality decreases), the edges remain intact which is the prime factor for frame isolation. The outcome from the last cascaded filter then goes through the threshold finder block after which the output from both filter paths are fed to the decoder. The threshold path output serves as the limits for frame identification of signals from the other path.

### 5.3. Hybrid Approach

The hybrid design is a straightforward approach derived from the combination of the summation and delimiter designs. While the core rationale behind the summation approach is to elevate the received signal by means of aggregating signals from different filters, the delimiter aims to keep signal quality and yet efficiently recognize frames. Both schemes have their strengths and weaknesses as will be seen in Section 6. The hybrid approach attempts to benefit from both the delimiter and summation designs. Figure 5c shows the structural design of the hybrid approach. The upper part of the architecture represents the addition of filtered signals with different lengths with an extension in the lower part to determine the boundary limits for decoding.

### 5.4. Threshold Finder for Frame Detection

A key element in the proposed filter design is the threshold finder for frame detection. The development and design of the multi-level filters are to stabilize the received signals for easy elimination of undesired signals. We propose the use of a mean-based approach in determining the threshold instead of a constant value which produces practically unrealistic outcomes. The equation for the threshold finder is as follows:

(3) $$\begin{array}{c} threshold=\left(\frac{1}{P}\sum_{i=1}^{P}x_{i}\right)\;\times c \end{array}$$

where *P* is the number of sample points, $x_{i}$ is the input signal and *c* is the scaling factor. The scaling factor *c* is set to ensure that none of actual signal samples are removed by lowering the threshold.

**Figure 7:**
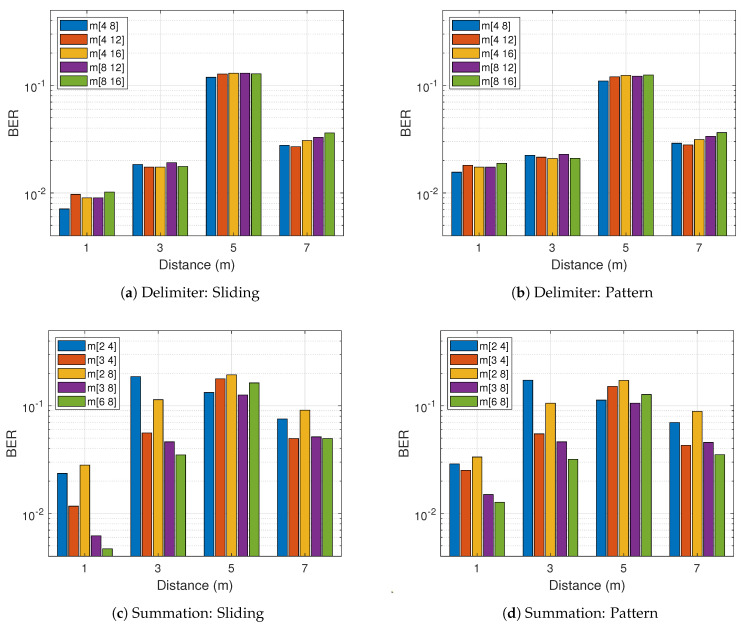
Experimental results for different filter constant *m* with sliding and pattern-based decoding.

**Figure 8:**
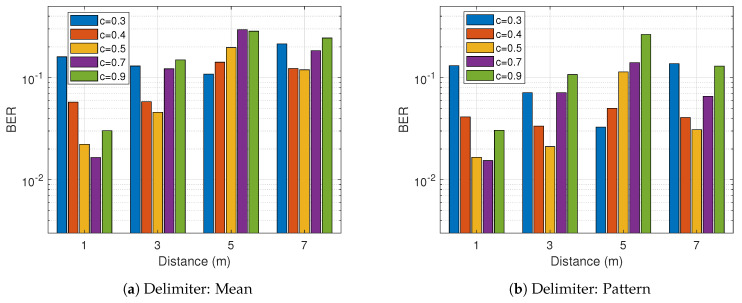

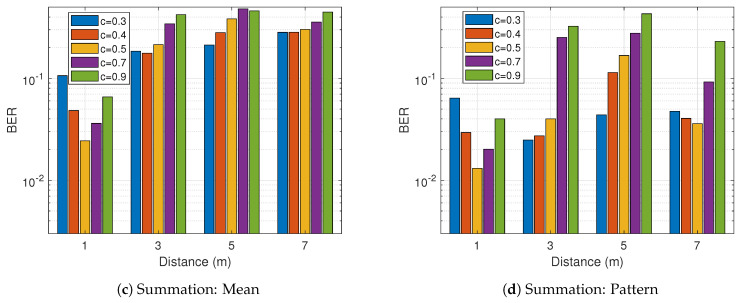
Experimental results for different threshold constant *c* with sliding and pattern-based decoding.

Selecting optimal configurations for *N*, *m* and *c* cannot be underestimated as they alter the operation design massively. From experimental observation, the optimal value for the filter length *N* equals half the symbol length where symbol length = sample rate/tag rate. The delimiter design is less affected by the choice of *m* provided the choice is multiple of 4. The optimal constants for *m* from Figure 7a,b are 4 and 8 for the delimiter approach because they are slightly better and cost-effective. On the other hand, the couple selection of 6 and 8 proves efficient for the summation approach demonstrated in Figure 7c,d. The constant factor *c* for the threshold finder upon several tests were evaluated at 0.5 as illustrated in Figure 8. The 0.5 chosen for the constant factor serves as the offset and it is stochastically valid at 50%.

## 6. Performance Evaluation

This section is categorized into four areas: configuration and setup, filter significance, indoor experimental analysis and hallway experimental analysis. Components, parameters and settings are explained in the first part whereas the essence or need of filter and for that matter, multi-level design is considered in the second part. Detailed analysis of the setup for good and bad circumstances are further investigated.

### 6.1. Setup and Configuration of Experiment

**Figure 9:**
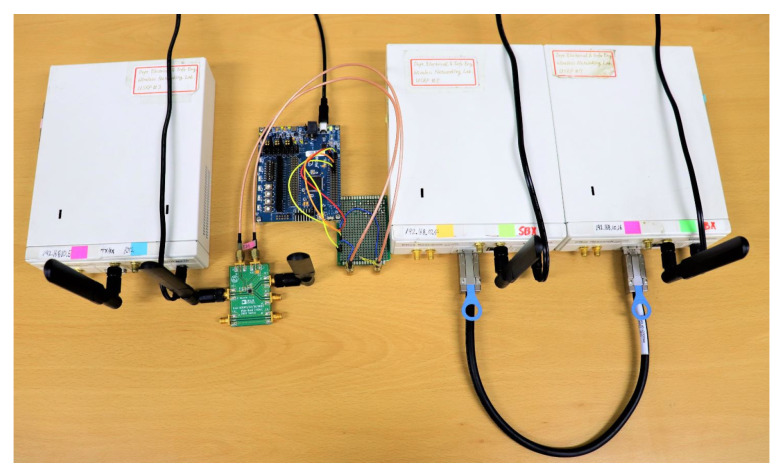
Hardware peripherals for testbed implementation.

The proposed multi-filter designs were implemented in MATLAB after raw signals are captured from the hardware peripherals. The physical components of the testbed are shown in Figure 9. A USRP N210 at channel 9 was used for WiFi signal generation and transmission. A receiver was implemented using two N210s (RX at channel 5 and channel 13) coupled together with an MIMO cable. A tag system is composed of an Igloo nano FPGA [29] for control signals and an RF switch (ADG902) [30] for reflecting/absorbing incident WiFi signals. GNU Radio [31], a set of tools for software defined radio (SDR) systems [32], was used in Linux Ubuntu OS for generating IEEE 802.11 g signals from the gr-ieee802.11 package [33]. The experiment was carried out in a line of sight scenario with two locations: office and hallway. Table 1 shows the parameters that are common to both experimental environments whereas specific parameters are outlined in detail during their analysis.

### 6.2. Significance of Filtering

**Figure 10:**
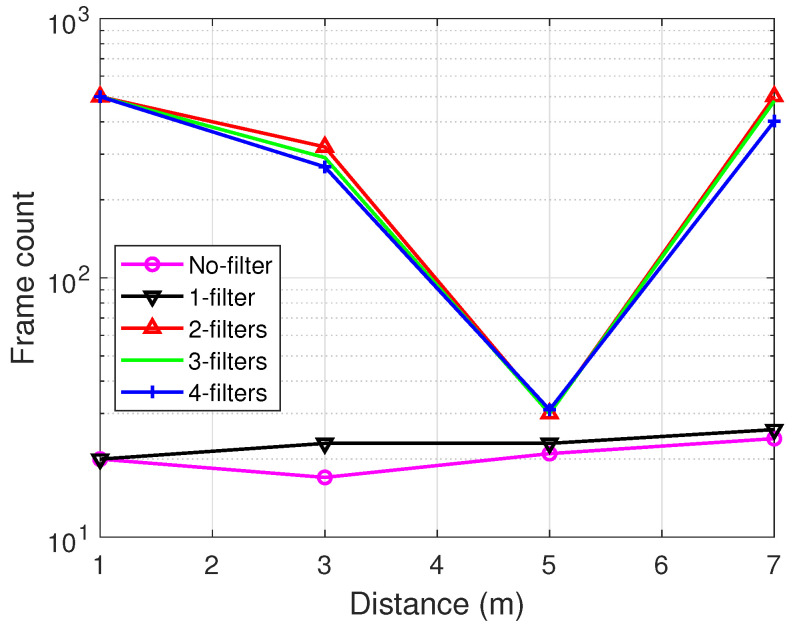
Number of frames extracted from received signal.

Before we move into the deep analysis of the proposed multi-filter design in comparison with the classical approach, it is imperative to validate the reason why the low-pass filter is important in the decoding process. The low-pass filter is purposefully meant to filter out signal fluctuations and provide smoothing for effective decoding. The smoothing effect in addition to noise removal improves frame identification and for that matter boosts the overall system performance. Basically, there are two reasons why filter(s) are required for data extraction: (1) adequate identification (detection) of frames and (2) efficient decoding in terms of BER performance. Figure 10 illustrates the number of detected frames for five different cases: no filter, one filter, two filters, three filters and four filters. The lowest frame count is witnessed when there is no filter used. However, a notable gap is observed when the filter is more than one except for distance 5 m (hallway), i.e., 5 m was a unique case of poor performance for the backscatter communication because the tag was far apart from both the receiver and the transmitter alike. Two reasons are given for the closeness with respect to the number of frames identified for no filter against filter designs: (1) only a small number of frames were distinct for decoding and (2) due to the nature of the frames identified, no filter performs a little below filter inclusion. The observation in Figure 10 underscores the superiority of utilizing more than one filter in view of the fact that a system should decode all frames transmitted.

**Figure 11:**
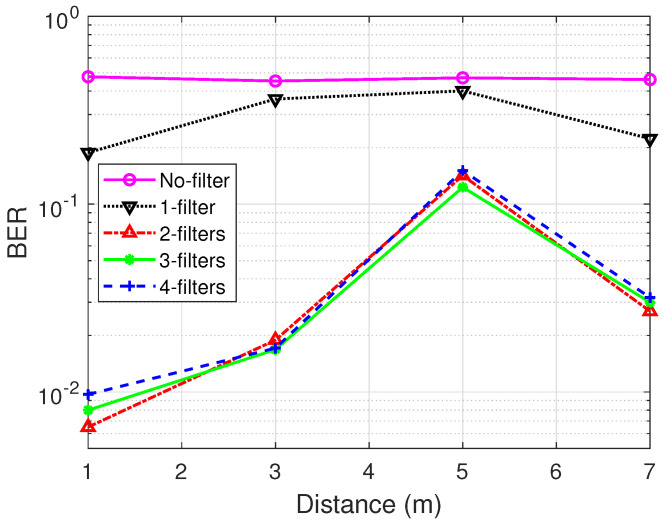
Bit-error-rate comparison of no filter, filter and multi-filter.

Figure 11 demonstrates the performance of no filter vs. filter designs with respect to BER. A similar trend as seen in frame count is seen where the BER results of the multi-filter designs outperform the no and one filter in that order. This concludes that for all intents and purposes, the inclusion of more filters is crucial for efficient decoding in backscatter communication.

It is of particular interest to note that there is no significant difference as the number of filters increases from two upwards even so in both figures. In Figure 11, however, the three-filter design proves slightly resilient especially at worse conditions (5 m) among the multi-filter designs. Therefore, our design under consideration for the following evaluation is incorporated with three filters.

### 6.3. Office Scenario

**Figure 12:**
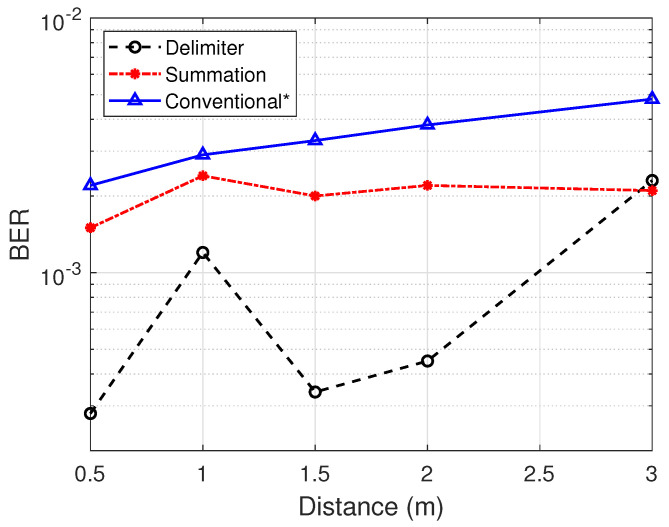
Bit-error-rate comparison with good environmental conditions in the office scenario.

The experiment inside the office room was characterized as a good channel environment due to the fact that (1) the transmission gain of the transmitter USRP is relatively higher, (2) the tag-to-transmitter distance is close and fixed and (3) there is less network traffic. The transmitter was positioned 50 cm away from the tag system while the receiver was placed at 0.5, 1.0, 1.5, 2.0 and 3.0 m away from the tag in the opposite direction. Tag data rates of 200 kbps and lower achieve BER of zeros for proposed designs. Figure 12 shows the results of multi-filter designs against the conventional approach for decoding backscattered signals with 1000 WiFi frames for each case at 500 kbps. The performance metric was BER against the receiver distance.

The conventional method considers the best threshold for each distance by means of trial and error. Selecting a constant threshold for the traditional approach will fail as the distance increases. Bear in mind that in the plot, the conventional method could not identify 1000 frames for all cases as the others could. It can be observed from Figure 12 that the performance of the proposed filters, i.e., summation and delimiter designs perform better than the conventional filter in all scenarios. The margin of BER increases at 2 m, demonstrating a huge performance difference between the filter designs to the tune of 42% and 88% enhancement for the summation and delimiter designs, respectively, compared to the conventional. The summation design marginally surpasses the performance of the delimiter design at 3 m. Figure 12 follows the normal trend with an increase in distance resulting in higher BER. However, the delimiter and summation approaches experience inconsistent results at 1 m, which is possibly due to unpredictable multipath reflections in the experimental locations.

### 6.4. Hallway Scenario

**Figure 13:**
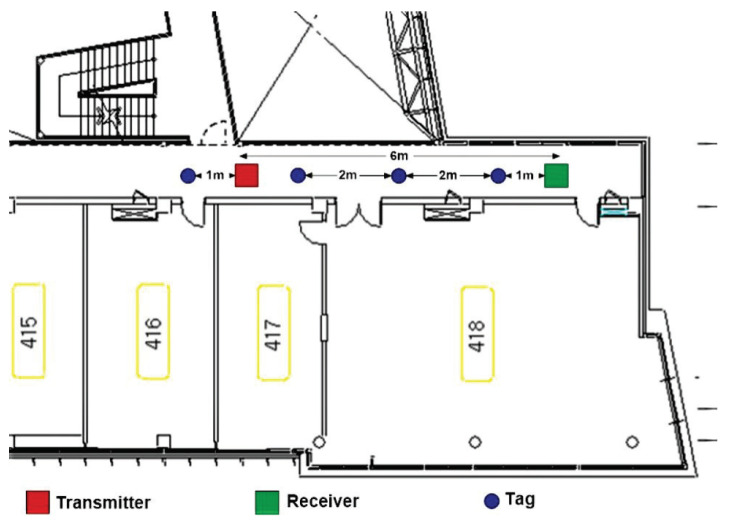
Hallway experimental setup.

Next, the experiment was carried on in the hallway of a building with relatively bad channel conditions such as lower transmission gain, varying tag-to-transmitter and tag-to-receiver distance and long distance of about 6 m between receiver and transmitter. The transmitter and receiver are fixed as shown in Figure 13 but the backscatter tag is initially positioned at 1 m away from the receiver in-between the transmitter. After every instance, the tag is moved 2 m from the previous step up until 7 m which is beyond the receiver by 1 m.

The BER was evaluated in combination with three different decoding algorithms to ascertain and prove the wide applicability and validity of our proposed schemes:

- *Mean-based decoding*: The arithmetic mean of the samples of a detected frame is used as the threshold to decide if a signal is deemed zero or one. In this context, since the reference signal is an FM0, the mean-based threshold is fixed to 0.5.
- *Sliding-based decoding*: A specific window size of a given buffer or array of signal samples is defined and the mean of window is used as the threshold for decoding.
- *Pattern-based decoding* [22]: The slope patterns of signals are uniquely identified and utilized for amplitude level classification.

**Figure 14:**
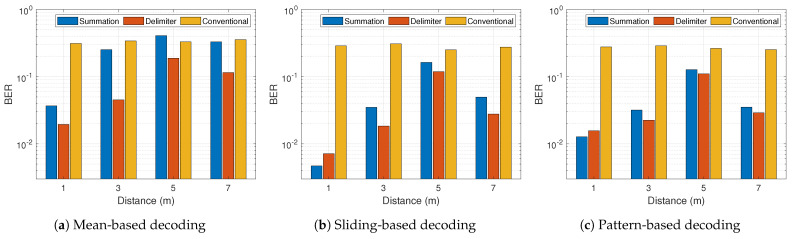
Bit-error-rate results for 100 kbps with three different decoding algorithms.

**Figure 15:**
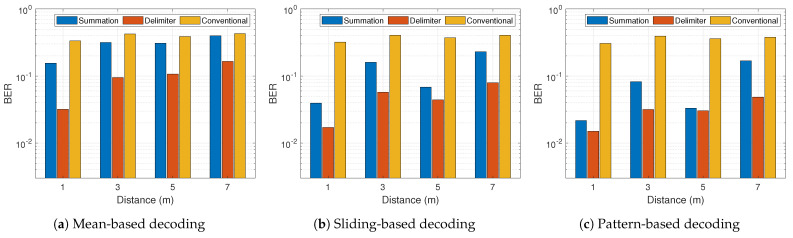
Bit-error-rate results for 500 kbps with three different decoding algorithms.

**Figure 16:**
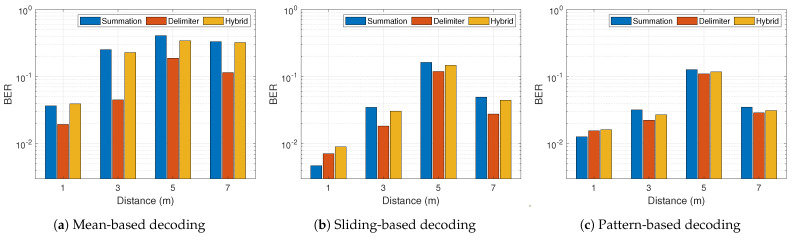
Results comparison for various proposed schemes at 100 kbps with different decoding algorithms.

In general from Figure 14 and Figure 15, the BER performance of both proposed schemes outperforms the conventional approach appreciably in all scenarios except Figure 14a at 5 m. At a data rate of 100 kbps, the sliding-based and pattern-based decoding with 1 m distance showed better BER for the summation multi-filter design in comparison with the delimiter design. This is prominent in Figure 14b,c. Other than that, the delimiter multi-filter design proves to be the most efficient approach in all conditions. Figure 14 and Figure 15 demonstrate the normal trend confirming the knowledge that higher data rate leads to higher BER. However, there was a peculiarity at 5 m, thus 500 kbps achieved lower BER than 100 kbps. The maximum BER improvement shows approximately 98.4% and 97.6% for summation and delimiter multi-filter designs, respectively, attained at a data rate of 100 kbps with sliding-based decoding algorithm placed at 1 m tag-to-receiver distance. Furthermore, Figure 16 depicts the clear distinction between the summation and delimiter designs juxtaposed with the hybrid approach. When the summation approach is better or close to the delimiter approach, the hybrid gives the best results evident from Figure 16b,c at 1 m and 7 m.

**Figure 17:**
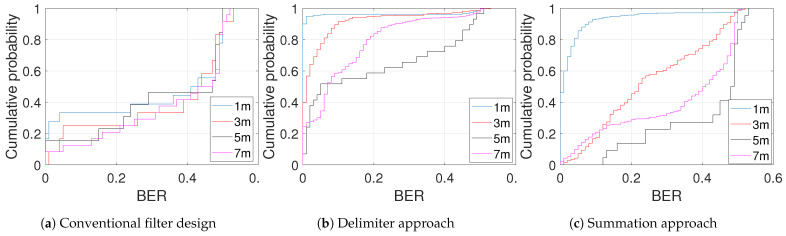
Cumulative probability distribution for various filter schemes: mean-based decoding at 100 kbps.

The cumulative distribution function (CDF) graphs for the multi-filter designs and the classical method are seen in Figure 17 with the mean-based decoding algorithm. Two features can be inferred from the given plots: (1) the amount of data points captured and (2) the cumulative probability of BER at each point. It is obvious from Figure 17a having the least amount of data implying the conventional filter design is unable to identify (detect) most of the frames while the proposed schemes (Figure 17b,c) do a significantly good job at detecting as many frames as possible. The CDF, in addition, reveals that at 1 m, the BER of 0.01 or fewer accounts for the probabilities of 0.946 and 0.8125 for delimiter and summation designs, respectively, while the conventional design attained a probability of 0.2778 at the same BER. This trend cuts across the increasing distance for the experiments 1 m, 3 m, 5 m and 7 m. This conclusion affirms the earlier statement that the proposed designs are more efficient especially the delimiter approach.

**Figure 18:**
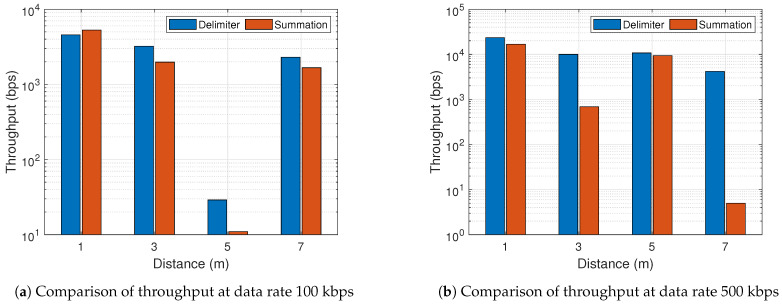
Results of throughput from the pattern-based decoding.

Figure 18 shows the results of throughput for the pattern-based decoding algorithm. The pattern-based decoding was selected for the throughput illustration because it demonstrates better performance compared to the other decoding algorithms. It is assumed that the tag transmits 50-bit packets back-to-back at the bit rate of 500 kbps during the first 1 ms of each WiFi frame, thus transmitting 10 packets per WiFi frame, which means the maximum achievable throughput is 50 kbps. The conventional design achieved near-zero throughput both 100 kbps and 500 kbps, so it is omitted from the figure. Thus, the proposed approaches prove evidently the huge performance gap over the conventional design. At a data rate of 100 kbps, the throughput at 1 m is slightly higher for the summation design than the delimiter agreeing with the exceptional instance for the BER performance explanation above.

**Figure 19:**
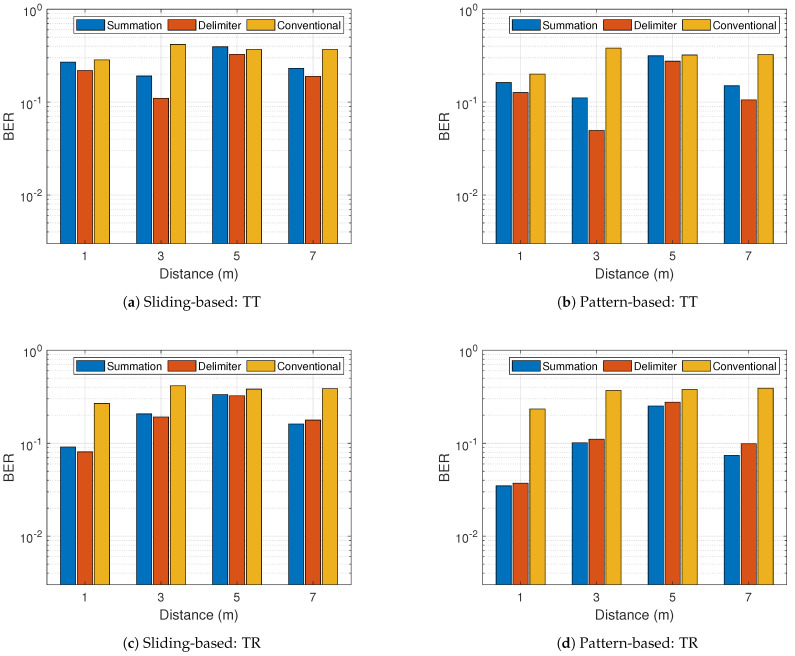
Bit-error-rate results with an obstacle at a data rate of 100 kbps (TT: obstacle between transmitter and tag, TR: obstacle between tag and receiver).

Lastly, two cases of obstacles in the hallway scenario are considered. Let TT be the instance where there is an obstacle between the transmitter and the tag and let TR be the scenario where an obstacle is placed between the tag and the receiver. The obstacle employed is a corrugated box made from 4 mm-thick corrugated cardboards; the overall dimension of the box is approximately 30 × 20 × 4 inches. The obstacle is erected to directly cover the full angle of line-of-sight. Figure 19 shows the BER results of TT and TR for the sliding-based and pattern-based decoding. The figure demonstrates that backscatter communication is highly affected by signal obstruction or blockage. Compared with the earlier results (no obstacle), BER is increased no matter the position of the obstacle. A critical observation from Figure 19 outlines the performance degradation is higher in TT (Figure 19a,b) than TR (Figure 19c,d). The logical reason behind the observation comes from the fact that in the case of TT, the tag receives attenuated carrier signals, thus triggering backscatter less while the tag in TR receives strong carrier signals first before backscattering.

### 6.5. Power Consumption and Processing Costs

While power consumption and processing costs remain intact in the tag, those of the receiver will increase due to increased complexity. For detailed analysis, the proposed multi-filter designs are implemented in an FPGA using Vivado Design Suite release 2020.1 from Xilinx [34]. This approach gives a solid estimate of logic counts which can predict the processing costs as well as power analysis by the tool, as we provide in Table 2. Hardware resources on an FPGA are indicated by the number of slices that FPGA has, where a slice is comprised of look-up tables and flip flops. Since approximating the realistic power consumption is dependent on the application design, complementary metal–oxide–semiconductor (CMOS) technology, voltage supply, clock frequency, the number of switching nodes, etc., we provide power values normalized by that of the single-filter design. The table shows that, while the number of slices of the multi-filter designs is 4–6× higher than the single-filter design, the increase of power consumption is 24–29% since the static common power factor is dominant. The authors in [35] demonstrate that an application-specific integrated circuit (ASIC) implementation of a moving average filter using 65 nm CMOS technology consumes 5.4 mW on average.

## 7. Conclusions

In this paper, we explored the significance of preprocessing filter for WiFi backscatter communication leading to a conventional single filter design, to be inefficient in noise removal and frame detection which causes significant degradation in performance. In an attempt to resolve this issue, we proposed multi-filter designs for WiFi backscatter communication with frequency-shift implementation. The proposed filters, i.e., the summation and delimiter designs, allow captured signals at the receiver to be separable by means of multi-level filtering and then input to a decoding algorithm. Our proposed designs from experimental results indicate a significant enhancement in throughput performance over the conventional approach. The BER performance of both multi-filter designs achieved better outcomes in various communication environments. Employing the proposed schemes with various decoding algorithms demonstrated a stable consistency of superior performance.

## Figures and Tables

**Table 1:** Experimental parameters.

Parameter	Value
WiFi frame transmission	Every 10 ms
WiFi frame duration	1.4 ms
WiFi frame size	1528 bytes
Data rate (MCS)	9 Mbps (QPSK 3/4)
Sampling rate	10 MHz
TX carrier frequency	2.452 GHz (channel 9)
RX carrier frequency	2.472 GHz (channel 13)
Frequency shift	20 MHz
Tag data rate	100, 500 kbps

**Table 2:** Synthesis results and power report of filter designs.

	Single Filter	Delimiter	Summation	Hybrid
Normalized power	1	1.242	1.260	1.286
Number of slices	214	1121	880	1191

## Data Availability

Not applicable.

## Abbreviations

The following abbreviations are used in this manuscript:

AP	Access Point
ASIC	Application-Specific Integrated Circuit
BER	Bit Error Rate
bps	bits per second
CDF	Cumulative Distribution Function
CMOS	Complementary Metal–Oxide–Semiconductor
COTS	Commercial Off-The-Shelf
CSI	Channel State Information
FPGA	Field Programmable Gate Array
FS	Frequency-Shifted
IoT	Internet of Things
MCS	Modulation and Coding Schemes
MIMO	Multiple Input Multiple Output
OFDM	Orthogonal Frequency-Division Multiplexing
RFID	Radio Frequency Identification
RSSI	Received Signal Strength Indicator
RX	Receiver
SDR	Software Defined Radio
TX	Transmitter
UAV	Unmanned Aerial Vehicle
USRP	Universal Software Radio Peripheral
